# Protective Effect of Natural Antioxidant Compounds on Methimazole Induced Oxidative Stress in a Feline Kidney Epithelial Cell Line (CRFK)

**DOI:** 10.3390/vetsci8100220

**Published:** 2021-10-08

**Authors:** Flavia Girolami, Alessia Candellone, Watanya Jarriyawattanachaikul, Giorgia Meineri, Carlo Nebbia, Paola Badino

**Affiliations:** Department of Veterinary Sciences, University of Torino, L. go Braccini 2, 10095 Grugliasco, TO, Italy; alessia.candellone@unito.it (A.C.); watanya.jarriyawattanachaikul@unito.it (W.J.); giorgia.meineri@unito.it (G.M.); carlo.nebbia@unito.it (C.N.); paola.badino@unito.it (P.B.)

**Keywords:** feline hyperthyroidism, methimazole, oxidative stress, quercetin, resveratrol

## Abstract

The treatment of choice for feline hyperthyroidism is the administration of the antithyroid drug methimazole. Both the endocrinopathy and the drug adverse reactions (e.g., hepatotoxicosis, gastrointestinal disorders, and renal injury) are partly due to oxidative stress and redox unbalance. This study investigated the free radical production and the impairment of the antioxidant barrier induced by methimazole in an in vitro model of feline renal epithelium. The protective effects of quercetin and resveratrol were also explored. CRFK cells were incubated with a methimazole concentration equivalent to the maximum plasma levels in orally treated cats (4 µM), in the presence or absence of either one of the two selected antioxidants at different time-points (up to 72 h). Cell viability, ROS production, GSH levels, and mRNA expression of antioxidant enzymes (i.e., CAT, SOD, GPx, and GST) were assessed. Methimazole impaired cell viability and increased ROS levels in a time-dependent manner. Similarly, GSH content and CAT, SOD, and GPx3 expression were higher compared with control cells. Such effects were significantly counteracted by quercetin. These results provide new insights about the mechanisms underlying the methimazole-related side effects frequently observed in hyperthyroid cats. They also support the use of quercetin in the management of feline hyperthyroidism.

## 1. Introduction

The most common endocrine disease in middle-aged and geriatric cats is hyperthyroidism (FHT), which is characterized by an excess of thyroid hormones, namely triiodothyronine (T3) and thyroxine (T4) [1]. The signs and symptoms usually comprise weight loss despite polyphagia, increased activity/restlessness, polyuria/polydipsia, an unkempt coat, and gastrointestinal signs, such as vomiting and diarrhea [2]. Some studies underlined the relationship between an excess of thyroid hormones and other diseases, such as chronic kidney failure, pulmonary hypertension, and chronic heart disorders [3,4,5]. The gold standard therapy for FHT is the treatment with radioactive iodine. However, its feasibility is reduced by practical considerations, such as lack of a convenient referral center with a radiation license, client fears about radiation or quarantine, or high initial cost. An alternative approach is surgical thyroidectomy, of which application is limited by concurrent geriatric problems that are likely to increase the risk of anesthesia-related complications [6]. Thus, pharmacotherapy with thyroid peroxidase inhibitors, so-called anti-thyroid drugs, is often the sole treatment option for FHT, and methimazole (MMI) is the drug of choice [7].

MMI belongs to the drug class of thionamides and prevents the synthesis of T4 and T3, blocking both iodide oxidation to iodine and iodination of tyrosine residues in the thyroid hormone precursor (thyroglobulin), which are performed by the hemoprotein thyroid peroxidase (TPO). A proposed theory affirms that the sulfur moiety of MMI might interact directly with the iron atom at the center of TPO heme molecule, inhibiting its ability to iodinate tyrosine residues [8]. MMI is usually administered by the oral route at a starting dose ranging from 2.50 mg to 5.0 mg per cat twice daily, depending on the severity of the disease. The twice daily regimen is preferred to the single administration of a higher dose because it induces less serious adverse reactions [2]. Indeed, several cases of MMI-induced organ injury, including renal impairment, are reported in the literature both in humans and in cats [9,10,11]. Even if the mechanisms of MMI-related toxicity are not entirely elucidated, some evidence underlines the possible involvement of reactive metabolite formation, oxidative stress induction, intracellular targets dysfunction, and immune-mediated toxicity [12,13].

Free radicals are highly reactive molecular species that attack important macromolecules (i.e., DNA, proteins, carbohydrates, and lipids), leading to cell damage and homeostatic disruption. The most important oxygen-containing free radicals are reactive oxygen species (ROS). Their generation and negative effects are counteracted by the organism antioxidant barrier, which includes the enzymes superoxide dismutase (SOD), catalase (CAT), and glutathione peroxidase (GPx), as a first line defense [14]. Oxidative stress originates from an imbalance between the production of ROS and the antioxidant capacities of cells and organs, playing a crucial role in many chronic diseases, including hyperthyroidism [15,16]. Indeed, SOD, CAT, and GPx activities are increased in both plasma and gland tissue in human patients affected by hyperthyroidism and treated with MMI [17,18]. Similarly, the redox status of hyperthyroid cats is impaired compared with both healthy animals and cats affected by chronic non-thyroidal diseases [19]. Moreover, the redox unbalance worsens in feline patients receiving MMI [20], confirming that oxidative stress is partly responsible for the endocrine disease as well as for the MMI-induced toxicity in both humans and cats.

In addition to endogenous antioxidant defense, exogenous antioxidants are substances that improve the immune function and balance the cellular oxidative status by scavenging the free radicals and by interrupting the lipid peroxidation process. Antioxidant phytochemicals are widely found in fruits (e.g., blueberries, raspberry, blackberry, apple, grape, and pomegranate), vegetables, cereal grains, and plants [21]. The main group is polyphenols that retain the highest antioxidant content and that act as free radical scavengers and metal chelators, thanks to their chemical structure consisting of one or more aromatic rings [22].

Natural antioxidants may also act by boosting the endogenous antioxidant system, and their protective role in several chronic diseases has been documented in humans and animals [23,24]. We have recently demonstrated that the dietary supplementation of an antioxidant mixture, containing quercetin (Q), resveratrol (R), curcumin, and vitamin E, counteracts both the oxidative stress and the side-effects elicited by MMI in hyperthyroid cats [20]. Based on the results of the clinical trial, the aim of the present study was to further investigate the role of each antioxidant in reducing the toxicity of MMI in a feline in vitro model, through the evaluation of ROS generation and of selected antioxidant parameters. As preliminary experiments on vitamin E and curcumin resulted in inconsistent results (data not shown), Q and R were employed. To mimic the in vivo scenario, MMI concentrations were selected based on the plasma levels of cats orally treated at a therapeutic dosage. As the kidney is a target organ of MMI-induced toxicity, an immortalized cell line derived from the renal cortex of a normal cat (CRFK) was used [25]. Although uncertainty has been suggested about the epithelial vs. mesenchymal phenotype of CRFK cells [26], they have been originally described as epithelial [25], and they are widely used accordingly [27,28].

## 2. Materials and Methods

### 2.1. Materials and Chemicals

The CRFK cell line was purchased from ATCC (American Tissue Culture Collection, Manassas, VA, USA). Q, R, 1-methyl-2-imidazolethiol (MMI); dimethyl sulfoxide (DMSO); Neutral Red (NR); 2′,7′-dichlorodihydrofluorescein diacetate (DCFH-DA); and all cell culture reagents were from Sigma-Aldrich/Merck (St. Louis, MO, USA). The reported chemical purities of Q (product n° 337951) and R (product n° R5010) were ≥95% and ≥99%, respectively. All of the materials for the quantitative RT-PCR (q-PCR) analysis (including RNA extraction and cDNA synthesis) were supplied by Bio-Rad (Valencia, CA, USA). The BCA Protein Assay Kit was purchased from Thermo Fisher Scientific (San Jose, CA, USA).

### 2.2. Cell Culture and Treatment

Cells were grown in MEM medium supplemented with 10% heat-inactivated FBS, 2 mM L-glutamine, non-essential amino acids, 1000 units/mL penicillin, 100 μg/mL streptomycin, and 0.25 μg/mL amphotericin B. The cells were maintained at 37 °C in an atmosphere of 95% relative humidity and 5% CO_2_. The cells were trypsinized every 3–4 days for sub-culturing. For the experimental treatments, they were plated, allowed to attach for 24 h, and tested at approximately 60% confluence.

For the viability assays, the cells were seeded into 96-well culture plates at a density of 6000 cells/well in 200 µL medium and treated as explained below. To determine the concentrations of the antioxidants to be used in the co-incubation experiments, the cells were treated with increasing concentrations of Q (0.125–50 μM range) or R (0.12–50 μM range) for 24 and 48 h. To test the ability of the selected compounds to counteract MMI toxicity, the cells were exposed to MMI in the presence or absence of either Q (3 and 6 μM) or R (2 and 4 μM) for 24, 48, and 72 h. The concentrations of MMI (2 and 4 µM) were chosen based on the mean (2 µM) and maximum (4 µM) plasma levels attained in cats orally treated with the thionamide drug at a therapeutic dosage to reproduce the in vivo scenario [29].

For the ROS production assay, CRFK cells were seeded into 96-well fluorescence measurement specific plates (Greiner Bio-One Italia S.r.l, Cassina de Pecchi, Italy) at a density of 6000 cells/well in 200 µL medium. To test the ability of the selected antioxidants to counteract MMI-induced ROS production, the cells were exposed to MMI (4 µM) in the presence or absence of either Q (6 μM) or R (4 μM) for 24, 48, and 72 h.

For the assessment of gene expression and GSH levels, the cells were seeded in 6 cm dishes at a density of 8000 cells/dish and incubated with MMI (4 μM) in the presence or absence of either Q (6 μM) or R (4 μM). Gene expression was assessed after 4, 8, 16, and 24 h; the GSH levels were measured after 4, 8, 24, 48, and 72 h.

The assays performed in 96-well plates (i.e., viability and ROS production) included six replicates for each experimental condition, while experiments in 6 cm dishes (i.e., gene expression and GSH levels) were performed in duplicate. All experiments were conducted independently at least three times.

All chemicals were dissolved in DMSO (used as control) at a final concentration in the growth medium not exceeding 0.1% (*v*/*v*).

### 2.3. Cell Viability Assay

The viability of cells exposed to all of the tested substances was evaluated by the Neutral Red Uptake (NRU) assay according to the protocol outlined by Repetto et al. (2008) [30]. The absorbance values were measured at 540 nm with a microplate reader. Cell viability was expressed as a percent relative to control cells (0.1% DMSO).

### 2.4. Reactive Oxygen Species (ROS) Production Assay

At each time point, the cells were washed with PBS and 200 µL of 10 mM DCFH-DA solution was added into each well. The plates were covered with aluminum foil to avoid light interferences and incubated at 37 °C and 5% of CO_2_ for 30 min. Then, the DCFH-DA solution was removed, and the cells were washed twice with ice-cold PBS. Finally, 100 µL of ice-cold PBS was added, and the resulting fluorescence intensity was immediately measured using fluorescence spectroscopy at λex = 485 nm and λem = 530 nm. The data were normalized to the number of viable cells and measured with the NR assay performed in parallel on the same set of cells. The ROS production was calculated according to the following formula:

ROS = (% fluorescence intensity)/(% cell viability)

The method has been validated for cells exposed for 24, 48, and 72 h to increasing concentrations (2, 4, 6, and 8 µM) of menadione, chosen as a gold standard for ROS production (Supplementary Figure S1).

### 2.5. Determination of Reduced Glutathione (GSH) Content

At each time point, cells were collected using a rubber tipped cell scraper and centrifuged at 200× *g* for 5 min at room temperature. After pellet suspension in 0.1 M phosphate buffer pH 7.4, containing 0.1 M Tris acetate, 0.1 M KCl, 1 mM EDTA, and 18 μM butylated hydroxytoluene, the cells were lysed by four cycles of freezing–thawing (fresh frozen in liquid nitrogen and thawed at 37 °C), followed by ten cycles of 10 s of sonication on ice. The homogenate was centrifuged at 17000× *g* for 15 min at 4 °C, and the supernatant was stored at −80 °C until analysis. The protein concentrations were measured by BCA Protein Assay Kit. Reduced GSH content was determined with dithio-bis-nitrobenzoic acid (DTNB) on deproteinized samples as described elsewhere [31]. The results were expressed as μg of GSH per mg of protein.

### 2.6. Quantitative RT-PCR (q-PCR) Assays

Total RNA was isolated using the PureZOL™ RNA Isolation Reagent according to the manufacturer’s protocol. The RNA purity and quantity were evaluated by absorbance readings using the NanoDrop ND-2000 spectrophotometer (Thermo Fisher Scientific, Illkirch Cedex, France). The ratio of the optical densities measured at 260 and 280 nm were >1.9 for all RNA samples. One milligram of total RNA was reverse transcribed into cDNA using iScript™ cDNA Synthesis Kit, according to the manufacturer’s instructions, in a final volume of 20 µL. Sufficient cDNA was prepared in a single run to perform the q-PCR experiments for all of the selected genes.

Primers for CAT, SOD, GPx1, GPx3, GPx4, GSTA2, GSTM3, and GSTP1 were designed on *Felis catus* GenBank and Ensembl mRNA sequences using Primer 3 Software (version 3.0, Applied Biosystems, Foster City, CA, USA), while primers for tryptophan 50-monooxygenase activation protein zeta (YWHAZ) were from Penning et al. (2007) [32] Oligonucleotides were designed to cross the exon/exon boundaries to minimize the amplification of contaminant genomic DNA and were analyzed with the NetPrimer tool (available at http://www.premierbiosoft.com/netprimer/index.html, accession date: 1 January 2021) for hairpin structure and dimer formation. Primer specificity was verified with BLAST analysis against the genomic NCBI database. Table 1 summarizes the primer information, including sequences, gene accession numbers and amplicon sizes.

Each primer set efficiency comprised between 95% and 100%. YWHAZ was selected as the reference gene since its expression was not affected by any of the treatments, and it is reported as one of the most stable reference genes in feline kidney tissue [32]. q-PCR reactions were performed on 500 ng of cDNA in a final volume of 20 μL consisting of the 1× iTaq SYBR Green Supermix with ROX and an optimized concentration of each primer set (150 or 500 nM). PCR amplification was run on an ABI 7500 Real-time PCR System (Applied Biosystems) using 96-well optical plates under the following conditions: 30 s at 95 °C for polymerase activation, and 40 cycles of 15 s at 95 °C and 60 s at 60 °C. Each reaction was run in triplicate, and a no-template control was included using water instead of cDNA. The modulation of gene expression was calculated with the 2^−ΔΔ*C*t^ method, and data were expressed as fold-change compared with control samples [33].

### 2.7. Statistical Analysis

All values are shown as mean ± SEM of at least three independent experiments. The normal distribution of data was assessed according to the D’Agostino and Pearson normality omnibus test. Significant differences among groups were evaluated by one-way analysis of variance (ANOVA), followed by the Dunnett’s or Sidak’s post hoc tests. Differences were considered statistically significant when the two-sided *p* value was <0.05. Data analysis was performed with the GraphPad Prism 7.03 software (Graph Pad Software, San Diego, CA, USA).

## 3. Results

### 3.1. Effects of Natural Antioxidants on MMI-Induced Cytotoxicity

**Figure 1:**
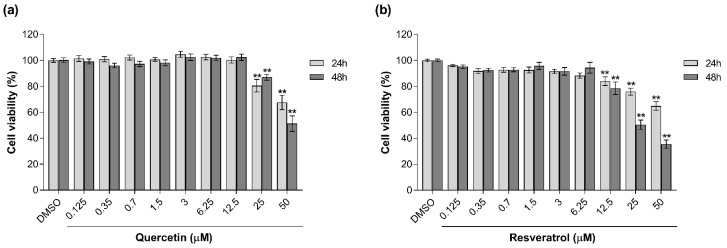
Effects of Q (0.12–50 μM) (**a**) and R (0.12–50 μM) (**b**) on CRFK cell viability after 24 and 48 h exposure measured with the NRU assays. Results are expressed as a percentage of viability compared with the solvent control (0.1% DMSO). Data are represented as mean ± SEM of three independent experiments and analyzed by one-way ANOVA followed by Dunnett’s test. Statistical differences with respect to the controls are indicated (** *p* < 0.01).

To investigate the ability of the selected antioxidants to counteract the toxicity induced by MMI in an in vitro model of feline kidney epithelium, CRFK cells were first exposed to each individual compound at different concentrations (range 0.125–50 μM) for 24 h and 48 h. The concentration response curves showed that both antioxidants significantly impaired cell viability (*p* < 0.01) only at very high concentrations (from 12.5 μM for R, and from 25 μM for Q) at both time-points (Figure 1). Based on such results, the concentrations 3 and 6 µM for Q and 2 and 4 µM for R were selected for use in the co-incubation assays.

**Figure 2:**
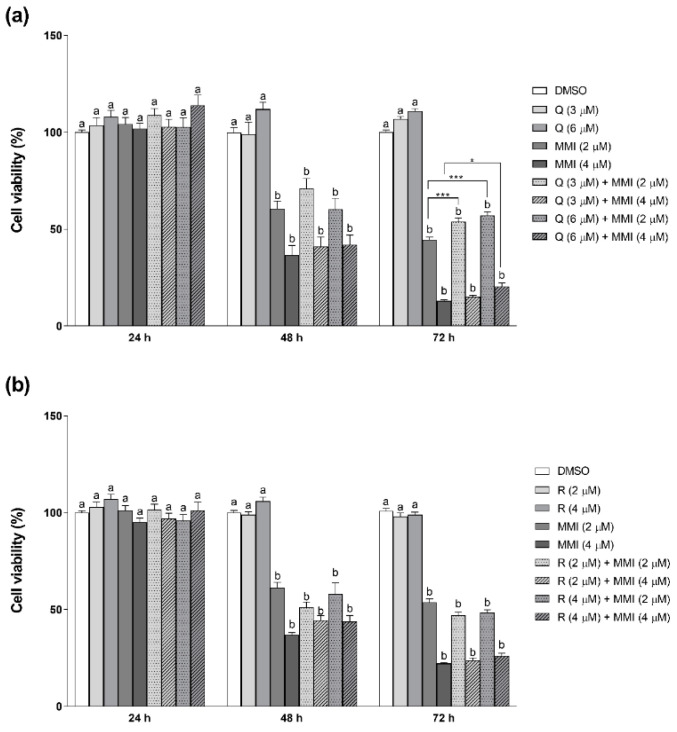
Effects of Q (**a**) and R (**b**) against MMI-induced toxicity in CRFK cells, measured with an NRU assay. Cells were exposed to MMI (2 and 4 μM) in the presence or absence of either Q (3 and 6 μM) or R (2 and 4 μM) for 24, 48, and 72 h. Results are expressed as a percentage of viability compared with the solvent control (0.1% DMSO). Data are represented as mean ± SEM of three independent experiments and analyzed by one-way ANOVA followed by Sidak’s test. Different letters indicate statistical differences compared with control cells (*p* < 0.05 or less). Statistical differences with respect to the cells treated with MMI alone are reported (* *p* < 0.05, *** *p* < 0.001).

As shown in Figure 2, both concentrations of MMI significantly reduced CRFK cell viability starting from 48 h (*p* < 0.01), with the maximum effect (80%) at 72 h at 4 μM concentration. At both tested concentrations, Q was able to significantly (*p* < 0.01) counteract the cytotoxicity elicited by 2 μM MMI at 72 h by approximately 25%. A similar effect was evident at the same time-point treating cells with Q 6 μM in the presence of the highest concentration of MMI (4 μM). Conversely, R did not protect CRFK cells from MMI cytotoxicity at any concentrations and any time-points.

### 3.2. Effects of Natural Antioxidants on MMI-Induced ROS Production

**Figure 3:**
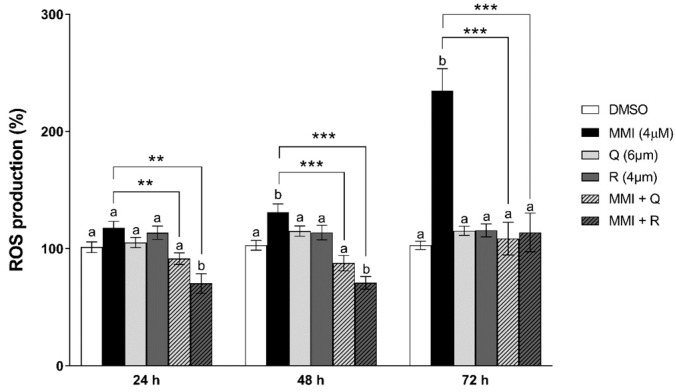
Effects of MMI, Q, and R on ROS production in CRFK cells, measured with the DCFH-DA assay. Cells were exposed to MMI (4 μM) in the presence or absence of either Q (6 μM) or R (4 μM) for 24, 48, and 72 h. Results are expressed as a percentage of ROS production compared with the solvent control (0.1% DMSO). Data are represented as mean ± SEM of three independent experiments and analyzed by one-way ANOVA followed by Sidak’s test. Different letters indicate statistical differences compared with control cells (*p* < 0.05). Statistical differences with respect to the cells treated with MMI alone are reported (** *p* < 0.01, *** *p* < 0.001).

To determine if the MMI-induced impairment of cell viability and the protection afforded by Q are related to the oxidative stress phenomenon, the DCFH-DA assay was performed in CRFK cells for the detection of the intracellular production of ROS (Figure 3). Q and R, used at the highest concentrations employed in the cell viability assays (6 and 4 μM, respectively) did not enhance the genesis of free radicals compared with control cells at any time-points. Moreover, R even decreased ROS levels with respect to cells incubated with DMSO at 24 and 48 h. Conversely, MMI (4 μM) triggered a statistically significant increase in ROS with respect to controls at 48 and 72 h by approximately 30% and 100%, respectively. Such an effect was significantly counteracted at both time-points by both Q and R, which prevent ROS levels from rising above control values. Moreover, both antioxidants reduced ROS production compared with MMI-treated cells already at 24 h.

### 3.3. Modulation of Reduced GSH Content by MMI and Antioxidants

Reduced GSH levels, measured at different time-points (4-8-24-48-72 h) upon incubation with MMI and/or Q or R, are reported in Table 2. With respect to control cells, R alone did not affect the GSH content at any time-points, while Q and MMI produced a statistically significant (*p* < 0.01) increase in GSH by approximately 2-fold at 8 and 24 h and at 48 and 72 h, respectively. In the presence of MMI, both antioxidants significantly (at least *p* < 0.01) enhanced GSH levels up to more than 2-fold compared with controls starting from 8 h. In the case of Q, such an increase was statistically significant (at least *p* < 0.05) also compared with MMI-treated cells at 8, 24, and 72 h.

### 3.4. Modulation of Gene Expression by MMI and Antioxidants

**Figure 4:**
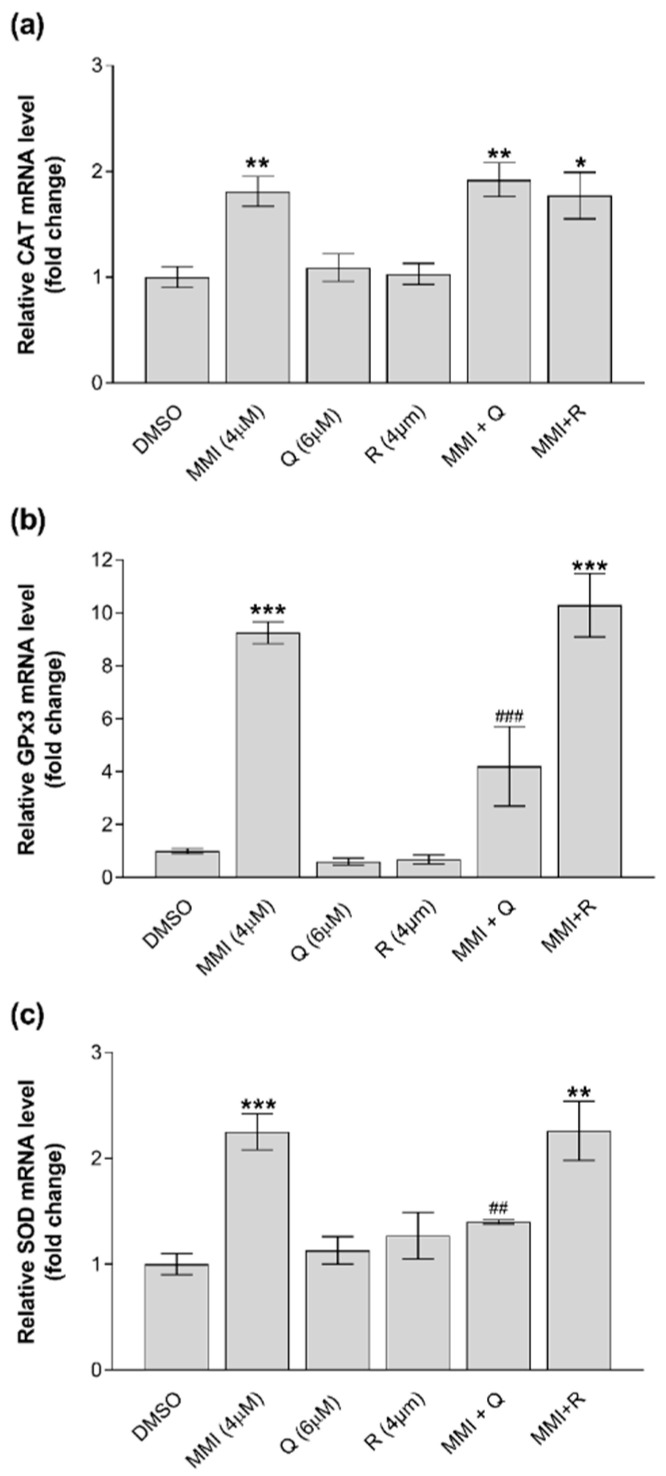
Gene expression modulation in CRFK cells treated with MMI (4 μM), Q (6 μM), or R (4 μM) or co-exposed to MMI (4 μM) + Q (6 μM), and MMI (4 μM) + R (4 μM) for 24 h. The mRNA expression levels of CAT (**a**), GPx3 (**b**) and SOD (**c**) were determined by quantitative RT-PCR. Results are expressed as a fold change compared with the solvent control (0.1% DMSO). Data are represented as mean ± SEM of three independent experiments and analyzed by one-way ANOVA followed by Sidak’s test. Statistical differences with respect to the controls (* *p* < 0.05, ** *p* < 0.01, *** *p* < 0.0001) and with respect to the cells treated with MMI alone (^##^ *p* < 0.01, ^###^ *p* < 0.001) are indicated.

To study the possible mechanisms responsible for the protective effects observed against MMI-induced cytotoxicity and oxidative stress exerted by Q and R in CRFK cells, the modulation of genes encoding for the antioxidant defense system (i.e., CAT, SOD, and GPx) and for the enzymes involved in GSH conjugation (i.e., GST isoforms) was evaluated upon incubation with MMI and/or Q or R at different time-points (4, 8, 16, and 24 h). The q-PCR results showed that CRFK cells express all of the investigated genes at appreciable levels, with SOD being the most expressed and with the isoform 3 of GPx (GPx3) being the least expressed (data not shown). GPx1, GPx4, and all GST isoforms (i.e., A2, M3, and P1) were not modulated at any time-points by any of the treatments, neither by the single molecules nor under co-incubation conditions (data not shown). The remaining tested genes (i.e., CAT, GPx3, and SOD) were modulated only after 24 h (Figure 4). In all cases, MMI significantly (*p* < 0.05 or less) upregulated gene expression to a variable extent, while Q or R alone did not affect the mRNA levels. The exposure to MMI alone increased the expression of CAT and SOD by approximately 2-fold and that of GPx3 up to 9-fold. The positive modulation of GPx3 and SOD elicited by MMI was significantly (*p* < 0.01) counteracted only by Q, while CAT upregulation was not reduced by any of the antioxidants.

## 4. Discussion

Chronic renal disease is an adverse effect that can be observed in hyperthyroid cats receiving MMI. On the one hand, kidney impairment is often a consequence of a long-lasting undiagnosed and untreated thyrotoxicosis that damages renal tissue leading to fibrosis [10]. On the other hand, MMI and its metabolites may lead to histological alterations that comprise glomerulosclerosis, edema and atrophy of the distal and proximal tubules, and cellular discontinuity of the Henle’s loop [34]. Such damage is likely associated with an increase in oxidative stress markers (ROS and lipid peroxidation) that is not compensated for by the antioxidant system [35]. Thus, the use of exogenous antioxidants could be attractive to counterbalance the generation of free radicals and to reduce the cytotoxicity induced by MMI. Since hyperthyroidism itself is associated with oxidative stress and redox impairment [15,16], the antioxidant supplementation is expected to be beneficial also for the improvement of the thyroid function. Even if the available information is scant, promising results have already been reported in hyperthyroid cats treated with MMI [20].

The results of our study demonstrated in a species-specific in vitro model that MMI at concentrations equivalent to those obtained in plasma from treated cats [29] impaired the viability of kidney cells by inducing ROS generation. In the only available study, the cytotoxic effect of MMI associated with oxidative stress has been reported in rat cultured hepatocytes, even if at different time-points (up to 3 h) and using MMI at a much higher concentration (10 mM vs. 4 µM) [36]. Our findings demonstrate for the first time that MMI, already at therapeutic concentrations, is able to induce oxidative stress in cat cells, supporting the hypothesis of its role in the development of adverse reactions. Moreover, the oxidative stress induced by the long-term MMI treatment might play a role in the progressive increase in the prevalence of large thyroid tumors, intrathoracic thyroid masses, and suspected thyroid carcinoma in hyperthyroid cats, as described by Peterson et al. (2012) [37]. Indeed, oxidative stress is a well-known carcinogenic factor that could be counteracted by the antiproliferative and antitumorigenic action exerted by antioxidant compounds. The rise in ROS levels recorded at 48 and 72 h following MMI treatment in CRFK cells was accompanied by a two-fold increase in GSH content. A different effect has been described in rat hepatocytes, where a remarkable decrease in reduced GSH was associated with a significant increase in the oxidized form (GSSG) [36]. One of the possible explanations of such a discrepancy could be the different time-points at which the GSH measurement was performed (starting at 4 h in CRFK cells and up to 3 h in hepatocytes). Indeed, pro-oxidants typically trigger a biphasic response consisting of an acute GSH-depletion followed by a later restoration toward or even over the baseline levels (“rebound effect”) [38]. Nevertheless, the involvement of other factors cannot be excluded, such as the amount of GSH used to neutralize the oxidative stress (depending on both the *noxa* and the tissue), and activity of GSH transferase enzymes. The impact of MMI on the cellular antioxidant system was also confirmed by the modulation of the antioxidant enzymes. Indeed, CRFK cells treated with MMI displayed a significant increase in the mRNA expression of CAT, SOD, and GPx3 at 24 h. Interestingly, GPx3 was the most modulated gene. This is line with its central role in renal tissue, where this selenoprotein isoform is primarily synthetized and then actively secreted into the blood [39]. Indeed, some studies highlighted the modulation of GPx3 in the course of kidney damage in different species [40,41]. To the best of the authors’ knowledge, this is the first report describing the transcriptional effects of MMI in vitro. The only available data refer to rat models of hypothyroidism obtained through the continuous administration of MMI. Santi et al. [42] investigated the antioxidant enzyme activity in the kidney of such rats and demonstrated that SOD but not CAT was upregulated by MMI.

Although Q and R have been proven to have antioxidant capacities in several in vitro and in vivo models, no data are available about their effect in MMI-treated cells. Thus, the present study reports for the first time the ability of both antioxidants to neutralize the ROS production triggered by the antithyroid drug. Surprisingly, the decrease in ROS levels produced by R is not accompanied by a reduction in cell death, as for Q, suggesting that the latter has a higher protective effect. Such a discrepancy could be explained by the greater modulation of the antioxidant system induced by Q in response to MMI treatment. Both compounds increased the GSH content in the presence of MMI at all time-points starting from 8 h; however, only Q was able to significantly restore SOD and GPx expression to control levels. In this respect, the counteraction by Q of MMI-induced SOD, but not CAT, activity has been reported in the liver of rats experimentally treated to develop hypothyroidism [42]. Although Q and R are both dietary polyphenols exhibiting anti-inflammatory and antioxidant properties, differences in the ability to neutralize oxidative and/or inflammatory stimuli have already been reported in several disease scenarios [43,44,45]. Even if no explanations have been provided so far about such a discrepancy, the different chemical structures of the two compounds may play an important role. Indeed, Q is a flavonoid while R is a non-flavonoid polyphenol, and these two classes are reported to act differently on antioxidant enzymes [46,47].

In conclusion, we report here that MMI, the drug of choice for FHT treatment, impairs feline kidney cell viability, increasing ROS generation and modulating the antioxidant system. Q, but not R, can efficiently counteract the MMI-induced cytotoxicity by enhancing the GSH content and by restoring both the ROS levels and the expression of the antioxidant enzymes (i.e., SOD and GPx3). Taking into account the limitations of an in vitro experimentation, the results reported here support the dietary supplementation of antioxidants, including Q, in the management of hyperthyroid cats to ameliorate the redox status [20], suggesting that it may also preserve the kidney function of the patients. There are other potential limitations in our study that could be addressed in future research. First, we explored the effects of each individual antioxidant compound. It would be of interest to assess if the different molecules could have synergistic effects that may potentiate the protective efficacy against MMI-induced prooxidant activity. Second, further investigations are warranted to unravel the molecular mechanisms of MMI cytotoxicity and its counteraction by natural antioxidants, including the evaluation of antioxidant enzyme activities as a complementary tool of the gene expression modulation. Finally, a similar experimental study performed on feline thyroid cells could provide useful information about the potential role of antioxidants in the amelioration of the thyroid function in the course of MMI therapy.

## Figures and Tables

**Table 1:** Primers for quantitative RT-PCR analysis.

Gene	Accession No.	Sequence (5′–>3′)	Amplicon Size (bp)
CAT	XM_003993157.4	FW: ACGCCTGTGTGAGAACATTG REV: TCACTGAAGTTCTTGACCG	82
GPx1	XM_004001361.5	FW: GGGCATCAGGAGAACGCTAA REV: CGCCATTTACCTCGCACTTC	115
GPx3	XM_003981387.5	FW: AACGGGGAGAAAGAGCAGAA REV: TTCCCAGAAGAGGCGGTTAG	96
GPx4	XM_023242756.1	FW: TCACCAAGTTCCTCATTGACA REV: TAGAGGTAGCAGGGCAAGTC	100
GSTA2	XM_011282429.3	FW: ATGTGGAAGAGCTTGACCCC REV: CGGGAGGGAGATTGCTGATT	84
GSTM3	XM_003990413.5	FW: CCGTTTTGAGGCTTTGGAGA REV: TTGGGCCATCTTGTTGTTGA	85
GSTP1	XM_023240130.1	FW: TCGCAGCAAATACATCACCC REV: GTCTCGAAAGGCTTCAGGTG	96
SOD1	XM_006935922.3	FW: CATCATTGGCCGCACGAT REV: ATGACACCACAAGCCAAACG	114
YWHAZ	XM_006943327.4	FW: GAAGAGTCCTACAAAGACAGCACGC REV: AATTTTCCCCTCCTTCTCCTGC	115

**Table 2:** Reduced GSH content in CRFK cells treated with MMI (4 μM), Q (6 μM), or R (4 μM) or co-exposed to MMI (4 μM) + Q (6 μM), and MMI (4 μM) + R (4 μM) after 4, 8, 24, 48, and 72 h.

	GSH Content (µg GSH/mg Protein)				
	4 h	8 h	24 h	48 h	72 h
**DMSO**	129.3 ± 25.2	106.2 ± 9.8	122.6 ± 19.3	85.0 ± 14.1	73.88 ± 12.0
**MMI**	118.9 ± 15.4	145.3 ± 10.7	176.1 ± 20.1	151.6 ± 10.5 *	154.4 ± 15.5 *
**Q**	117.4 ± 23.9	215.0 ± 26.4 *	226.2 ± 16.8 *	119.8 ± 13.6	100.7 ± 10.1
**R**	109.1 ± 19.1	130.1 ± 10.7	142.84 ± 18.6	99.1 ± 12.8	94.8 ± 17.6
**MMI + Q**	120.8 ± 24.4	220.8 ± 5.4 *^#^	268.6 ± 4.9 *^#^	208.5 ± 23.5 *	224.8 ± 24.7 *^#^
**MMI + R**	117.5 ± 16.6	200.1 ± 19.2 *	219.7 ± 7.0 *	192.3 ± 13.2 *	197.2 ± 12.0 *

Data are expressed as mean ± SEM of three independent experiments and analyzed by one-way ANOVA followed by Sidak’s test; * *p* < 0.01 or less as compared with DMSO, ^#^ *p* < 0.05 or less compared with MMI.

## Data Availability

All of the data analyzed during this study are included in this published article and in its Supplementary Materials.

## Supplementary Materials

The following are available online at https://www.mdpi.com/article/10.3390/vetsci8100220/s1, Figure S1: Effects of menadione (M) on ROS production in CRFK cells, measured with the DCFH-DA assay.
