# Supplementary material for: Protective Effect of Natural Antioxidant Compounds on Methimazole Induced Oxidative Stress in a Feline Kidney Epithelial Cell Line (CRFK)
Source: Vet Sci. 2021 Oct 8;8(10):220. doi: 10.3390/vetsci8100220 (PMC8541200; doi:10.3390/vetsci8100220)
Supplement: Supplementary file 1 [file vetsci-08-00220-s001.zip › vetsci-1387924-supplementary.pdf]

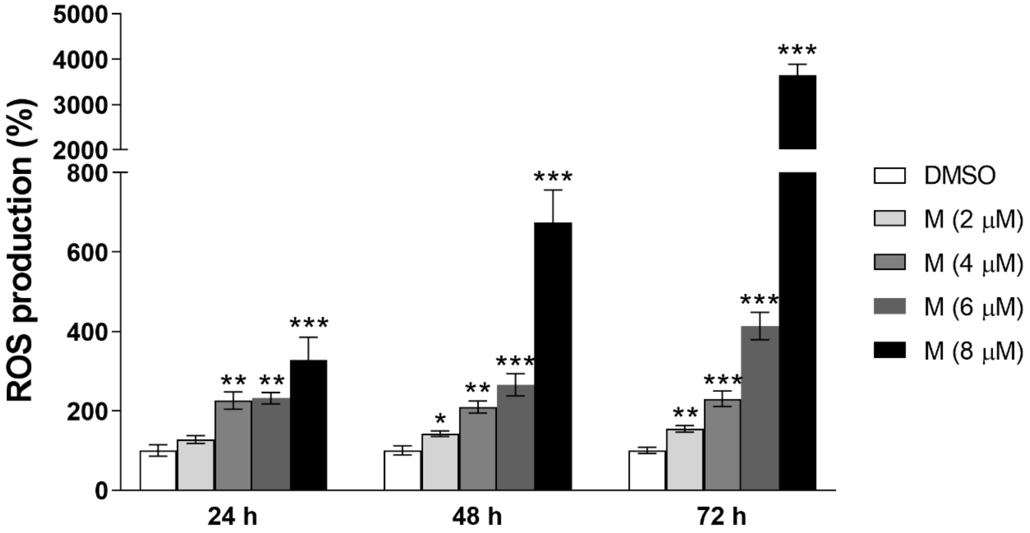

Figure S1. Effects of menadione (M) on ROS production in CRFK cells, measured with the DCFH-DA assay. Cells were exposed to menadione (2, 4, 6 and 8 μM) for 24, 48, and 72 h. Results are expressed as a percentage of ROS production compared to the solvent control (0.1% DMSO). Data are represented as mean ± SEM of three independent experiments and analyzed by one-way ANOVA followed by Dunnett's test. Statistical differences with respect to the controls are indicated (* $p < 0.05$; ** $p < 0.01$; *** $p < 0.001$).
